# Management and clinical outcomes of cardiogenic shock in King Abdulaziz University Hospital: A retrospective study

**DOI:** 10.15537/smj.2023.44.5.20220704

**Published:** 2023-05

**Authors:** Hamza L. Fida, Mohammed A. Qutub, Alaa S. Algazzar, Abdulrahman S. AlDharrab, Terad A. Alnajjar, Anas M.A. Andijani, Ahmad M. Taiyeb

**Affiliations:** *From the Faculty of Medicine (Fida, AlDharrab, Alnajjar, Andijani, Taiyeb), and from the Department of Medicine (Qutub, Algazzar), King Abdulaziz University,; and from the Department of Cardiology (Aljazzar), Ahmed Maher Teaching Hospital, Jeddah, Kingdom of Saudi Arabia.*

**Keywords:** shock, cardiogenic, dobutamine

## Abstract

**Objectives:**

To assess frequencies of various management approaches in cardiogenic shock (CS) and their clinical outcomes. Cardiogenic shock is a state of organ hypoperfusion and hypoxia caused by cardiac failure.

**Methods:**

In this retrospective record review, we assessed the presentations, vital signs, laboratory readings, and treatments for 188 consecutive CS inpatients from 2010-2021. Patients were labeled as “ischemic CS” or “non-ischemic CS” based on the occurrence of myocardial infarction as the precipitating cause, and “post-operative CS” if they had undergone cardiac surgery. In-hospital mortality was the primary endpoint of the study.

**Results:**

We identified 118 (62.8%) ischemic, 64 (34%) non-ischemic, and 6 (3.2%) postoperative CS patients. The study population had a high mortality rate (85.1%). Logistic regression analysis revealed that dopamine (*p*=0.040) and epinephrine (*p*=0.001) were independent predictors of mortality, while dobutamine (*p*=0.004) and digoxin (*p*=0.044) associated with increased survival. No significant association with mortality was found between either PCI or IABP. No significant difference in mortality was observed between CS subgroups.

**Conclusion:**

Variations in outcomes occurred with different medications. Mortality was higher in patients receiving dopamine or epinephrine and lower in those receiving dobutamine or digoxin. Implementation of clinical trials for investigation of the mortality benefit observed with dobutamine can serve towards formulation of new guidelines for improvement of CS mortality rates.

**S**hock is a critical condition of circulatory failure that results in insufficient oxygen supply to satisfy cellular metabolic needs and oxygen consumption requirements, resulting in life-threatening hypoxia of cells and tissues.^1^ Cardiogenic shock (CS), a subtype of circulatory shock, is a complex and hemodynamically diverse state characterized by low cardiac output that leads to decreased end-organ perfusion and hypoxia, often accompanied by multisystem organ failure.^2,3^

Acute myocardial infarction (MI) with left ventricle dysfunction is the most prevalent cause of CS.^2^ Similarly, CS is the most commonly reported cause of mortality in patients presenting with MI.^4^ Other reported causes of CS include cardiomyopathy (tako-tsubo or hypertrophic), as well as acute presentations of myocarditis, pericarditis, and valve regurgitation.^2^ Furthermore, the overall in-hospital mortality rate of CS is greater than 50%.^5,6^

The prevalence of CS in intensive care units (ICUs) and intensive cardiac care units ranges from 14% to 16%, illustrating the immense burden on healthcare facilities caused by CS.^5,7^ A previous study conducted in France in 2016 reported a significant increase in the prevalence of CS over a 15-year study period, with a younger and more critically ill patient population presenting most recently, and a noticeable decline in mortality over time due to a universal improvement in patient management.^8^ A more recent study conducted in Germany expressed the different treatment modalities used and clinical outcomes achieved in non-ischemic CS versus ischemic CS, revealing significant differences in management decisions and a poorer prognosis for non-MI-related CS.^9^

As reported by the American Heart Association (AHA), only a few evidence-based therapeutic interventions have been shown to improve patient outcomes in CS;^10^ moreover, there are insufficient studies that are carried out in Saudi Arabia which discuss the different management modalities and clinical outcomes of CS. Therefore, this study aimed to describe the rates of utilization of different treatment approaches in patients admitted to King Abdulaziz University Hospital (KAUH) with CS in different clinical contexts, and to compare their effects on patient survival rates.

## Methods

Approved by the KAUH research ethics committee (reference no. 703-20), this retrospective record review was carried out in June 2021 in KAUH, a tertiary healthcare facility located in Jeddah, Saudi Arabia. The study was carried out in the Department of Medicine, Coronary Care Unit (CCU) and ICU. The study procedures were performed following the ethical principles of the Helsinki Declaration based on the guidelines published by the World Medical Association. The requisite for informed consent from each patient was waived on account of the observational nature of the study.

Consecutive CS patients between January 2010 and April 2021 were identified using International Classification of Diseases (ICD) -10 codes associated with circulatory shock (R57.0, R57.8, and R57.9), and electronic patient records were accessed to confirm that CS was the principal diagnosis. The CS was defined according to the European Society of Cardiology (ESC) guidelines as a drop in systolic blood pressure (SBP) below 90 mmHg that is unresponsive to fluid resuscitation, a requirement of vasopressor administration to maintain SBP above 90 mmHg, and clinical or laboratory findings consistent with hypoperfusion (for example, oliguria, mental confusion, and elevated serum lactate).^11^

All CS patients of ≥18 years of age were included in this study, while patients with coexisting other types of shock were excluded. The participants were then organized into the following groups for comparison: ischemic, non-ischemic, and postoperative CS. Hereby, ischemic CS refers to patients who developed CS as a complication of an acute MI, with or without ST-segment elevation; postoperative CS refers to patients who developed CS after undergoing cardiac surgery; and non-ischemic CS refers to all other CS patients, including those who presented with CS at admission.

The information obtained from patient records included sociodemographic information and known medical conditions, as well as the dates of admission and discharge. The data on baseline vital signs and lowest blood pressure readings were collected for each patient, along with the most significant CS-related laboratory results obtained during their hospital stay (including highest serum lactic acid, highest creatinine, and highest hepatocellular enzymes). Hypotension was defined as SBP below 90 mmHg or diastolic blood pressure below 60 mmHg.^12^ Tachycardia was defined as an increase heart rate above 100 beats per minute.^13^ Data on thrombolysis in MI (TIMI) flow grade was collected for participants who had undergone coronary angiography and defined according to the original publication from the AHA.^14^ Furthermore, left ventricular ejection fraction was recorded in patients who underwent echocardiography.

Treatments were classified into 3 groups: medical treatment (for example, vasopressors, inotropes, and anticoagulants), mechanical support (for example, intra-aortic balloon pump [IABP]), and revascularization procedures (percutaneous coronary intervention [PCI] and coronary artery bypass grafting [CABG]). Evaluation of the clinical outcome for each patient was then performed by documenting in-hospital mortality as the primary endpoint of the study.

### Statistical analysis

Patient data was entered into Microsoft Excel version 16 (Microsoft Corporation, Redmond, WA) and then analyzed using IBM Statistical Package for the Social Sciences version 21 (IBM Corp. Armonk, NY). Qualitative data was presented in frequency and percentage, while quantitative data was presented in mean and standard deviation; t-tests and chi-squared tests were used to assess the differences in patient characteristics, and Fisher’s exact test was used for situations with expected cell frequencies less than 5. Binary logistic regression analysis was used to determine independent predictors of in-hospital mortality. A *p*-value <0.05 was regarded as statistically significant. A total of 4.8% of data items were missing and omitted from the respective statistical analysis.

## Results

Between January 2010 and April 2021, 188 patients met our inclusion criteria and were enrolled in this study. The study population was comprised of 119 (63.3%) men and 69 (36.7%) women and had an average age of 64 years (standard deviation=14.3). Of these patients, 118 had presented with ischemic CS (62.8%), 64 (34%) presented with non-ischemic CS, and 6 (3.2%) had postoperative CS. The most common comorbidity in this cohort was diabetes mellitus (71.3%), followed by hypertension (64.9%), and heart failure (60.6%). In addition, only 9.6% of patients presented with systolic hypotension during admission (n=18), while 30.3% presented with diastolic hypotension at admission (n=57). Tachycardia at admission was present in 45.7% of patients (n=86). The detailed patient characteristics and objective findings are presented in Table 1.

**Table 1:** - Patient characteristics and objective findings.

Variables	All	Ischemic CS	Non-ischemic CS	Post-operative CS	*P*-value
Count	188	118	64	6	
***Demographics***					
Age in years	64.3 (14.3)	65.6 (12.7)	62.6 (16.8)	55 (12.7)	0.109
Male gender	119 (63.3)	83 (70.3)	33 (51.6)	3 (50.0)	0.024
***Known medical conditions***					
Diabetes mellitus	134 (71.3)	88 (74.6)	42 (65.6)	4 (66.7)	0.418
Heart failure	114 (60.6)	67 (56.8)	44 (68.8)	3 (50.0)	0.244
Hypertension	122 (64.9)	76 (64.4)	42 (65.6)	4 (66.7)	1.000
Arrythmia	36 (19.1)	21 (17.8)	14 (21.9)	1 (16.7)	0.821
Cardiomyopathy	15 (8.0)	6 (5.1)	9 (14.1)	0 (0.0)	0.131
Valvular disorder	44 (23.4)	25 (21.2)	16 (25.0)	3 (50.0)	0.226
Mitral regurgitation	28 (14.9)	19 (16.1)	8 (12.5)	1 (16.7)	0.739
Aortic regurgitation	13 (6.9)	5 (4.2)	6 (9.4)	2 (33.3)	0.021
Renal disease	78 (41.5)	44 (37.3)	34 (53.1)	0 (0.0)	0.013
Pulmonary disease	62 (33.0)	31 (26.3)	30 (46.9)	1 (16.7)	0.010
***Vital signs***					
HR at admission (BPM)	92.1 (27.1)	92.1 (28.1)	91.5 (26.3)	101.3 (13.0)	0.789
RR at admission (per minute)	25.3 (8.2)	25.4 (7.6)	25.5 (9.5)	20.3 (2.1)	0.466
O_2_ saturation at admission (%)	94.2 (6.9)	94.6 (6.1)	93.5 (8.1)	92.5 (11.0)	0.594
SBP at admission (mmHg)	120.9 (29.4)	121.7 (31.1)	118.1 (26.9)	137.8 (10.9)	0.397
Lowest SBP (mmHg)	92.5 (23.5)	93.4 (22.8)	90.5 (23.6)	94.5 (41.3)	0.779
DBP at admission (mmHg)	66.8 (18.8)	67.0 (19.6)	66.0 (16.7)	71.5 (27.2)	0.838
Lowest DBP (mmHg)	50.3 (16.2)	50.8 (16.5)	49.0 (14.2)	56.0 (31.1)	0.650
***Investigations***					
Lowest hemoglobin (g/dL)	8.8 (3.1)	9.0 (2.6)	8.6 (4.0)	7.9 (1.0)	0.605
Lowest LVEF (%)	32.2 (13.7)	31.0 (12.0)	34.5 (15.9)	30.0 (18.4)	0.284
Highest creatinine (µmol/L)	402.3 (265.5)	439.1(283.7)	347.6 (224.6)	270.3 (171.8)	0.039
Highest BUN (mmol/L)	28.4 (16.7)	26.9 (15.4)	32.3 (18.5)	16.8 (11.8)	0.025
Lowest platelet count (x109/L)	131.4 (107.0)	135.1 (118.9)	128.2 (85.0)	93.0 (67.2)	0.619
Highest lactic acid (mmol/L)	13.0 (35.4)	11.4 (23.6)	15.8 (49.0)	5.9 (1.3)	0.729
Highest PT (sec)	44.8 (142.9)	47.4 (179.3)	41.2 (30.0)	30.2 (7.5)	0.932
Highest PTT (sec)	83.4 (37.8)	85.2 (39.6)	79.2 (35.2)	91.5 (30.1)	0.518
Highest ALT (U/L)	887.2 (1697.5)	1134.3 (2043.8)	489.4 (719.0)	353.8 (493.4)	0.037
Highest AST (U/L)	2834.7 (4481.3)	3177.5 (4697.4)	1982.5 (3653.7)	5357.8 (6911.3)	0.091
***Outcome***					
In-hospital mortality	160 (85.1)	104 (88.1)	52 (81.3)	4 (66.7)	0.155

CS: cardiogenic shock, HR: heart rate, RR: respiratory rate, SBP: systolic blood pressure, DBP: diastolic blood pressure, LVEF: left ventricular ejection fraction, BUN: blood urea nitrogen, PT: prothrombin time, PTT: partial thromboplastin time, ALT: alanine aminotransferase, AST: aspartate aminotransferase. Age and measurements shown are represented as mean (standard deviation [SD]), and one-way ANOVA was used for statistical analysis. Gender, known medical conditions, and outcomes are represented as frequency (percentage within the CS subgroup); the statistical test used was the Chi-squared test

Regarding the utilization of treatment modalities, 115 patients in our study had received mechanical ventilation (61.2%), while 53 patients had undergone PCI (28.2%), and 37 had an IABP (19.7%) (Table 2). Moreover, 56 patients in this study underwent coronary angiography with a median of 2 diseased vessels, with the majority presenting with grade 0 TIMI flow (60.4%; n=32) and 50% achieved grade 3 flow after completing PCI (n=28). The mortality rate in the study population was 85.1% (n=160).

**Table 2:** - Medical management, mechanical supportive treatment, and interventions for cardiogenic shock patients.

Variables	All	Ischemic CS	Non-ischemic CS	Post-operative CS	*P*-value
Dopamine	94 (50.0)	61 (51.7)	31 (48.4)	2 (33.3)	0.707
Dobutamine	67 (35.6)	46 (39.0)	19 (29.7)	2 (33.3)	0.433
Norepinephrine	100 (53.2)	65 (55.1)	32 (50.0)	3 (50.0)	0.790
Epinephrine	83 (44.1)	56 (47.5)	25 (39.1)	2 (33.3)	0.528
Digoxin	24 (12.8)	9 (7.6)	13 (20.3)	2 (33.3)	0.011
Thrombolysis	13 (6.9)	12 (92.3)	1 (7.7)	0 (0.0)	0.097
IV Antiplatelet	32 (17.0)	17 (14.4)	15 (23.4)	0 (0.0)	0.197
Oral Antiplatelet	164 (87.2)	112 (94.9)	47 (73.4)	5 (83.3)	0.000
IV Anticoagulant	153 (81.4)	103 (87.3)	47 (73.4)	3 (50.0)	0.008
Oral Anticoagulant	19 (10.1)	11 (9.3)	6 (9.4)	2 (33.3)	0.197
IABP	37 (19.7)	36 (30.5)	0 (0.0)	1 (16.7)	0.000
Mechanical ventilation	115 (61.2)	78 (66.1)	35 (54.7)	2 (33.3)	0.122
PCI	53 (28.3)	51 (43.2)	1 (1.6)	1 (16.7)	0.000
CABG	20 (10.6)	15 (12.7)	3 (4.7)	2 (33.3)	0.044

CS: cardiogenic shock, IV: intravenous, IABP: intra-aortic balloon pump, PCI: percutaneous coronary intervention, CABG: coronary artery bypass grafting. Data are represented as frequency (percentage within the CS subgroup), statistical test used was Chi-squared test

Our analysis revealed that the rate of occurrence of ischemic CS was significantly higher in male patients than in female patients (*p*=0.024); however, gender had no significant impact on survival across all CS groups on both bivariate (*p*=0.741) and multivariate (*p*=0.175) analyses. Furthermore, our results show an expected higher frequency of use of both PCI and IABP in ischemic CS patients (both *p*<.001), despite subgroup bivariate analysis showing no significant improvement in mortality (*p*=0.752 and *p*=0.547).

Further, the adjustment for applicable confounders in a binary logistic regression model (Table 3) showed that dopamine (*p*=.040) and epinephrine (*p*=0.001) were both independent predictors of in-hospital mortality in this cohort. In contrast, the administration of either dobutamine (*p*=0.004) or digoxin (*p*=0.044) was associated with lower mortality. The addition of recorded laboratory values in the regression model showed that elevations in either creatinine (odds ratio [OR], 1.004; 95% CI, 1.001-1.008) or blood urea nitrogen (BUN) levels (OR, 1.053; 95% CI, 1.007–1.101) were significantly associated with higher mortality rates. It is also worth noting that there was no significant difference in mortality rates between the different groups of CS, as indicated by both bivariate (*p*=0.155) and multivariate (*p*=0.339) analyses.

**Table 3:** - Binary logistic regression model for prediction of in-hospital mortality.

Variables	Odds ratio	95% CI	*P*-value
Age	1.026	0.985–1.070	0.219
Gender	0.444	0.138–1.433	0.175
Diabetes mellitus	0.589	0.151–2.303	0.447
Hypertension	2.052	0.576–7.306	0.267
Pulmonary disease	0.324	0.086–1.222	0.096
Renal disease	1.000	0.292–3.425	1.000
Heart failure	1.009	0.280–3.629	0.990
Arrhythmia	1.230	0.265–5.699	0.791
Valvular disorder	0.258	0.070–0.954	0.042
Dopamine	4.346	1.068–17.675	0.040
Dobutamine	0.123	0.029–0.515	0.004
Norepinephrine	0.344	0.078–1.515	0.158
Epinephrine	16.216	2.987–88.044	0.001
Digoxin	0.277	0.054–0.959	0.044
Thrombolysis	0.328	0.027–3.962	0.381
IV antiplatelet	0.969	0.234–4.013	0.965
Oral antiplatelet	0.094	0.007–1.180	0.067
IV anticoagulant	1.125	0.290–4.370	0.864
Oral anticoagulant	1.300	0.228–7.416	0.768
Mechanical ventilation	2.505	0.728–8.620	0.145
IABP	1.220	0.138–10.810	0.858
PCI	1.324	0.180–9.720	0.782

IV: intravenous, IABP: intra-aortic balloon pump, PCI: percutaneous coronary intervention, CABG: coronary artery bypass grafting, CI: confidence interval

## Discussion

An important finding in our study was that dobutamine administration was significantly associated with a lower in-hospital mortality rate. Similar results were obtained in a multicenter randomized trial conducted in France, which stated that dobutamine is the first-line inotropic agent to be used when norepinephrine fails to restore circulation in cases of CS.^15^ This result is also supported by the conclusions of a multicenter cohort study in which the administration of dopamine was an independent predictor of mortality, while that of dobutamine and epinephrine were not.^16^ The last study is also the reason that the German-Austrian guidelines favor dobutamine over other inotropic agents.^17^

Dobutamine is an inotrope that functions via a predominant beta-1 adrenergic receptor activation mechanism (increasing inotropy, chronotropy, and reducing left ventricular filling pressure).^18^ It also has a minor impact on alpha-2 and beta-2 adrenergic receptors, which results in vasodilation; therefore, the combined effect is an increase in cardiac output and a decrease in systemic vascular resistance with or without a slight drop in blood pressure.^18^ This effect necessitates caution when administering dobutamine in a hypotensive patient and explains the need for combination therapy with vasopressors such as norepinephrine. The administration of different catecholamines simultaneously and the unique circulatory situation of each patient may influence our findings; therefore, we suggest further studies and clinical trials to better assess the relationship between dobutamine and survival in patients with CS.

In the ischemic CS subgroup, there was a significant higher rate of use of IABP, despite regression analysis showing no effect on patient mortality. The IABP is still commonly used in many facilities around the world despite the results of multiple studies confirming the lack of improvement in mortality rate in patients with ischemic CS when applying IABP.^3,19,20^ This is possibly because the international guidelines have documented the use of IABP to improve blood pressure and perfusion of coronary arteries, explaining the widespread use of IABP as a standard practical procedure despite contradictory reports.^19^ Furthermore, the widespread use of thrombolytics and PCI in combination with IABP can change the accuracy of the impact of IABP alone.

We concluded from our analysis that epinephrine had significantly increased patient mortality, and this is congruent with a meta-analysis conducted in 2018 which stated that epinephrine increases the mortality rate by three-fold.^21^ Additionally, a review of articles conducted in 2020 also confirmed an increase in mortality rate following the administration of epinephrine.^22^ This may result from a concomitant increase in the oxygen demand of cardiac tissue, exacerbating myocardial damage.^23^ However, we were limited in quantifying due to the wide confidence interval generated.

Further, our analysis revealed that dopamine was associated with higher mortality rates. Other studies have shown similar results regarding this correlation; it was hypothesized that the increase in cardiac output caused by dopamine increases strain on cardiac muscle.^24^ Additionally, dopamine increases the risk of cardiac arrhythmia more notably than other catecholamines.^22,25^ However, a significant limitation we faced was the inability to narrow down dopamine as the cause of arrhythmia developing during hospital care, as numerous catecholamines and other medications were concomitantly administered to each patient.

### Study limitations

The inherent limitations of this study are related to its observational nature, which restricts our ability to identify true causality and eliminate persisting confounders. Moreover, underuse of the ICD-10 code for CS and poor documentation resulted in a smaller, more critically ill patient population with some missing vital sign recordings and laboratory readings, thereby limiting our identification of independent predictors of mortality and restricting the use of subgroup multivariate analysis. Furthermore, the manual review of patient records might have produced errors in the classification of patients or missed secondary diagnoses, which could interfere with our results.

In conclusion, this study had shown that significant differences in patient outcomes were observed when different medications were administered. The mortality rate was higher in patients receiving dopamine or epinephrine and lower in those receiving dobutamine or digoxin. And even though no such association was established between IABP or PCI with mortality, the underlying cause may be related to the critical condition of our patient sample.

Despite the limitations of this retrospective study, it sheds light on the characteristics of a sample of the local CS population and confirms the high mortality rate. This study can also be a first step towards formulation of new guidelines for improvement of CS patient outcomes; we hereby strongly recommend further investigating the mortality benefit of dobutamine as a first- or second-line inotrope in local CS patients through clinical trials. Furthermore, future investigations should focus on non-ischemic CS patients, as there has been limited literature published on this topic.
